# Circulating Bile Acids Profiles in Obese Children and Adolescents: A Possible Role of Sex, Puberty and Liver Steatosis

**DOI:** 10.3390/diagnostics10110977

**Published:** 2020-11-20

**Authors:** Martina Montagnana, Elisa Danese, Alice Giontella, Sara Bonafini, Marco Benati, Angela Tagetti, Andrea Dalbeni, Paolo Cavarzere, Rossella Gaudino, Mairi Pucci, Gian Luca Salvagno, Franco Antoniazzi, Giuseppe Lippi, Claudio Maffeis, Cristiano Fava

**Affiliations:** 1 Section of Clinical Biochemistry, Department of Neuroscience, Biomedicine and Movement Science, University of Verona, 37134 Verona, Italy; elisa.danese@univr.it (E.D.); marco.benati@univr.it (M.B.); mairipucci@yahoo.it (M.P.); gianluca.salvagno@univr.it (G.L.S.); giuseppe.lippi@univr.it (G.L.); 2 “General Medicine and Hypertension” Unit, Department of Medicine, University of Verona, 37134 Verona, Italy; alice.giontella@gmail.com (A.G.); bonafinisara@gmail.com (S.B.); angela.tagetti@libero.it (A.T.); andrea.dalbeni@aovr.veneto.it (A.D.); cristiano.fava@univr.it (C.F.); 3 Department of Surgery, Dentistry, Paediatrics and Gynaecology, University of Verona, 37126 Verona, Italy; paolo.cavarzere@aovr.veneto.it (P.C.); rossella.gaudino@univr.it (R.G.); franco.antoniazzi@univr.it (F.A.); claudio.maffeis@univr.it (C.M.)

**Keywords:** bile acids, children, endothelial function, hypertension, NAFLD, obesity, steatosis

## Abstract

Background. Childhood obesity is becoming a major health issue and contributes to increasing the risk of cardiovascular disease in adulthood. Since dysregulated metabolism of bile acids (BAs) plays a role in progression of obesity-related disorders, including steatosis and hypertension, this study aimed to investigate BAs profiles in obese children with and without steatosis and hypertension, as well as exploring the interplay between BAs profile and vascular function. Methods. BAs concentrations were quantified with liquid chromatography-tandem mass spectrometry in 69 overweight/obese children and adolescents (mean age, 11.6 ± 2.5 years; 30 females). Liver steatosis was defined with abdomen ultrasonography, whilst hypertension was defined according to the current European guidelines. Vascular function was assessed with ultrasound technique, by measuring carotid intima media thickness (cIMT) and common carotid artery distensibility (cDC). Results. Total and individual glycine-conjugated BAs concentrations were found to be significantly higher in males compared to females, as well as in pre-pubertal compared to pubertal stage (*p* < 0.05 for both). No difference in BAs concentration was observed between hypertensive and normotensive subjects. Total BAs and glycine conjugated BAs were significantly higher in participants with steatosis compared to those without (*p* = 0.004 for both). The values of total glycine-conjugate acids were positively correlated with cDC and this association remained significant in linear regression after adjusting for sex, age, pubertal stage, body mass index and aspartate aminotransferase. Conclusion. The results suggest a possible role of BAs in the pathogenesis of liver and/or vascular damage in children and adolescent. Further studies are hence needed to validate these preliminary findings.

## 1. Introduction

The prevalence of overweight and obesity continues to increase worldwide, since these conditions represent one of the leading health issues in both childhood and adulthood [1,2]. Overweight and obese subjects are known to have increased risk of developing nearly every chronic condition (cardiovascular diseases, diabetes, dyslipidemia, cancer) [1,2]. Obese children are also more likely to maintain an unhealthy weight later in life, thus becoming obese or overweight [3], and thus displaying a higher risk of developing cardiovascular disease in adulthood [4].

Non-alcoholic fatty liver disease (NAFLD), a definition which encompasses a broad spectrum of pathologies ranging from fatty liver alone to steatohepatitis with fibrosis and even cirrhosis, is increasingly found in obese children and adolescents, thus representing the most frequent form of chronic liver disease in this population [5]. Nearly 40–70% of obese children have hepatic steatosis, which is universally considered a prevalent risk factor for hyperlipidaemia, insulin resistance and cardiovascular complications [6].

In light of all these considerations, the identification of biomarkers which could help accurately predicting the progression of these obesity-related disorders in childhood shall be seen as a primary healthcare goal.

Primary bile acids (BAs), cholic acid (CA) and chenodeoxycholic acid (CDCA), are endogenous steroid molecules synthesized in the liver from cholesterol, and stored in gallbladder in conjugated form (with either taurine or glycine) for facilitating their water solubility [7]. After nutrient ingestion, these compound are secreted within the duodenum, where they play a major role in regulating lipid metabolism [8], glucose absorption and promoting the digestion of dietary fat and fat-soluble vitamins [9]. These molecules are then bio-transformed in the colon into secondary BAs (e.g., deoxycholic acid—DCA, ursodeoxycholic acid—UDCA, and lithocholic acid—LCA) by gut microbiota, so that nearly 95% of BA can be reabsorbed in ileum and transported back to the liver, where they are re-conjugated and re-excreted into the bile through enterohepatic circulation. Only a minimal amount (~5%) of BAs is hence lost in the feces [10]. Although the concentration of BAs is typically low in peripheral blood under normal (e.g., steady-state) conditions, circulating BAs levels can rise or decrease, even substantially, in several hepatic and non-hepatic diseases [11,12].

BAs are bioactive molecules which function by activating a family of receptors, known as bile acid-activated receptors (BARs), expressed throughout the body in many different cells. Although participation in digestion and absorption of dietary fats and liposoluble vitamins seems to be the main physiological role of BAs [13], several studies showed that these compounds are involved in a vast array of other metabolic pathways [14].

Dysregulated BAs metabolism is hence suggested to play a crucial role in the pathogenesis of dyslipidemia, fatty liver disease, diabetes, obesity, and atherosclerosis [15]. Serum BAs levels also increase during NAFLD progression to fibrosis, thus representing a putative noninvasive biomarker of pediatric NAFLD progression [16].

We then hypothesized that serum BAs could be differently represented in obese children according to the presence of steatosis or hypertension and could be associated with either blood pressure (BP) or vascular elasticity and morphology. Therefore, this exploratory study aimed to investigate BAs profile in obese children with and without obesity-related complications, as well as to explore the relation between BAs profile and markers of early atherosclerosis

## 2. Materials and Methods

Sixty-nine consecutive overweight/obese children and adolescents (mean age, 11.6 ± 2.5 years; 30 females) were recruited from October 2012 to September 2014 from the “Paediatric Obesity Outpatients Unit” of the University Hospital of Verona and the “Local Health Unit n. 20” of Verona. Inclusion criteria were: age between 5–18 years; overweight or obesity (i.e., BMI ≥ 85th and 95th percentile for sex and age, respectively) [17]. The WHO reference for BMI was used for categorizing children within overweight and obese groups [18]. Body weight and height were measured with the patient wearing light clothes. Body weight was measured on a calibrated balance and height with a calibrated stadiometer. BMI was calculated as weight in kg divided by the square of height in m. Expert pediatricians defined the pubertal status based on Tanner stages, so that children with a score ≥3 were classified as pubescent.

Exclusion criteria were hepatic or renal chronic diseases, malignancies, diabetes mellitus, lipid-lowering or antihypertensive therapy, and secondary causes of obesity. The study was approved by the Ethical Committee of the University Hospital of Verona (project identification code: CE n. 2218, 26 September 2012), and written informed consent was obtained from all participants’ parents, in accordance with local laws and regulations.

During the visit, BP was measured with a semiautomatic oscillometric device (TM-2551, A&D instruments Ltd., Abingdon Oxford, UK) 3 times, 3 min apart with the patient lying supine for at least 10 min before the first measurement in a room with controlled temperature (22–24 °C). The mean value of 3 clinostatic measurements was calculated and considered for z-score and percentile calculation. BP levels were then confirmed with measurement in sitting position using an oscillometric device and auscultatory method. All values derived from BP measurements were transformed in z-score and percentile, according to normative values [19,20]. The 95th percentile of office and ambulatory BP measurements was used as cut-off for hypertension, according to the current European guidelines [19].

The presence of significant liver steatosis was defined according to ultrasonographic characteristics by an experience sonographer using abdomen US (ACUSON S2000TM system, Siemens, Erlanger, Germany). Diffuse liver hyperechogenicity relative to the kidneys, ultrasonography beam attenuation, or poor visualization of intrahepatic vessel borders and diaphragm were suggestive of hepatic steatosis [21,22]. According to previously evidence data, ultrasonography was found to have 80% sensitivity and 86% specificity for detecting moderate to severe steatosis in children [21,22].

cDC was calculated as: DC = ΔA/(A × ΔP), where A is the diastolic lumen area, ΔA is the stroke change in lumen area and ΔP is pulse pressure (PP). Changes in diameters were detected using ultrasound B-mode image sequences of right and left common carotid arteries acquired at different steps and analyzed with the aforementioned automatic system [23]. The relative z-score and percentile were calculated according to reference values [24].

Fasting blood samples were collected in the morning, between 8:00 and 9:00 A.M. Subjects were asked to abstain from strenuous exercise and avoid consuming caffeine-containing beverages within the 12 h before visit and venous sampling. Laboratory measurements, including fasting plasma glucose (FPG), insulin, total cholesterol, high density lipoprotein (HDL)-cholesterol, triglycerides, AST, ALT and gamma-glutamyl transferase (GGT) were measured with standardized laboratory methods on routine clinical chemistry instrumentation (Cobas 8000, Roche Diagnostics GmbH, Mannheim, Germany).

BAs concentrations were assayed in serum according to a previously published technique [25]. Briefly, separation and quantification of individual BAs was performed on an Acquity UPLC I-Class System coupled with a Xevo TQ-S micro tandem mass spectrometry detector (Waters Corporation, Milford, MA, USA) operating in electrospray negative ionisation mode (ESI-). Investigated BAs (n = 15) were the following: tauroursodeoxycholic acid (TUDCA), taurocholic acid (TCA), glycoursodeoxycholic acid (GUDCA), glycocholic acid (GCA), taurochenodeoxycholic acid (TCDCA), taurodeoxycholic acid (TDCA), cholic acid (CA), ursodeoxycholic acid (UDCA), glycochenodeoxycholic acid (GCDCA), hyodeoxycholic acid (HDCA), glycodeoxycholic acid (GDCA), taurolithocholic acid (TLCA), chenodeoxycholic acid (CDCA), glycolithocholic acid (GLCA), deoxycholic acid (DCA). BAs concentrations lower than the lower limit of quantitation LOQ (5 ng/mL for each bile acid) were imputed as 5/sqrt(2) ng/mL [26].

### Statistical Analysis

Serum BA concentrations are reported as median and interquartile range (IQR). The concentrations of the parameters tested were compared with Wilcoxon–Mann Whitney U test. The Spearman coefficient (rs) was calculated for quantifying the correlation between variables. Multivariate linear regression models were performed for testing if the association between BAs and markers of vascular damage remained significant after adjusting for age, sex, pubertal stage, BMI and AST.

All statistical tests were two-tailed and *p*-values of <0.05 were considered statistically significant. The SPSS Statistics package 21.0.0 (IBM SPSS, Armonk, NY, USA) was used for all analyses.

## 3. Results

The general characteristics of the 69 obese subjects included in this study are shown in Table 1 and Table A1.

Since the values of some BAs were below the limit of quantitation (LOQ) of the assay, BAs detected in less than 75% of the samples (tauroursodeoxycholic acid—TUDCA, hyodeoxycholic acid—HDCA, glycolithocholic acid—GLCA) were excluded from our statistical analysis. Of the 15 serum BAs, a final number of 12 (CA, CDCA, DCA, glycocholic acid (GCA), glycochenodeoxycholic acid—GCDCA, glycodeoxycholic acid—GDCA, glycoursodeoxycholic acid—GUDCA, taurocholic acid—TCA, taurodeoxycholic acid—TDCA, taurochenodeoxycholic acid—TCDCA, hyodeoxycholic acid—HDCA, and UDCA) could be accurately quantified and were hence included in our analysis.

As shown in Table 2, total glycine-conjugated BAs concentrations were higher in male than in female obese children/adolescents (*p* = 0.005). All individual glycine-conjugated BAs (GCA, GCDCA, GDCA, GUDCA) were also found to be higher in male than in female obese children/adolescents (*p* < 0.05 for all).

Total glycine-conjugated BAs concentrations and GCA levels were higher in pre-pubertal than in the pubertal subjects (*p* = 0.02 and *p* = 0.03, respectively).

No significant difference in BAs concentration could be found between hypertensive and normotensive obese children/adolescents (Table A2).

Unlike these findings, total BAs and glycine-conjugated BAs were found to be significantly higher in subjects with ultrasonography (US) detectable steatosis than in those without (*p* = 0.004 for both) (Table 3).

Primary glycine-conjugated BAs concentrations (GCA and GCDCA) and primary taurine-conjugated cholic acid (TCA, taurocholic acid) were found to be higher in obese children/adolescents with steatosis than in those without (*p* < 0.05 for all).

### Correlation Analyses

No significant correlations were found between single or total BAs levels and body mass index (BMI) and between single or total BAs levels and age in the whole children population (Table 4).

After stratifying the population by sex, a significant negative correlation was found in boys between total BAs and age (r = −0.37, *p* < 0.05), as well as between glycine-conjugated BAs and age (r = −0.37, *p* < 0.05), whilst no significant correlations were observed in girls.

A significant positive correlation was found between glycine-conjugated BAs and aspartate aminotransferase (AST) (r = 0.35, *p* < 0.001), as well as between glycine-conjugated BAs and alanine aminotransferase (ALT) (r = 0.25, *p* < 0.05). All single glycine-conjugate BAs (GCA, GCDCA, GDCA, GUDCA) were positively correlated with both AST and ALT (r between 0.26 and 0.35, *p* < 0.05 for all).

Total glycine-conjugate BAs were positively correlated with common carotid artery distensibility (cDC) (measured as 10^−3^-Kpa and percentile) (r = 0.27, r = 0.26, *p* < 0.05). This association remained statistically significant and in multivariate linear regression analysis after adjusting for sex, age, pubertal stage, BMI, and AST [beta (SE): 0.268 (0.005), *p* = 0.032 for 10^−3^-Kpa; beta (SE): 0.291 (0.001), *p* = 0.020 for percentile]. Among single BAs, only GUDCA was positively correlated with cDC (both 10^−3^-Kpa and percentile) (r = 0.32, r = 0.31, *p* < 0.05) (Table 5).

GDCA and TDCA values were positively correlated with carotid intima-media thickness (cIMT) (both in absolute and percentile value) (r = 0.31 and r = 0.27, *p* < 0.05, for GDCA; r = 0.27 and r = 0.25, *p* < 0.05, for TDCA). All these correlations were no longer significant, however, after adjusting for sex, age, pubertal stage, BMI, and AST (we excluded from the adjustment ALT and GGT because these variables were in multi-collinearity with AST).

## 4. Discussion

An interesting aspect emerged from our investigation is instead that the BAs profile differs between the two sexes in obese children/adolescents. Unlike previous data in an unselected cohort of children [27], we found significant differences in glycine-conjugated BAs (GCA, GCDCA, GDCA and GUDCA) concentrations between males and females, with higher concentrations in male than in female obese children and adolescents.

Since our findings are in keeping with those previously published by Frommherz and colleagues in an adult cohort [28], we could speculate that the excess of adipose tissue, especially in the obese children’s liver, makes them more “metabolically similar” to adults, so that also the BAs pattern would become compatible to that observed in adults. In line with this hypothesis, obese children have higher estrogen values than normal-weight children [29] and display also an atherogenic dyslipidemia characterized by qualitative changes in LDL and HDL cholesterol [30], which could ultimately have an influence on BAs metabolism.

Estrogens are important regulators of BA synthesis and pool composition, and this is probably due to their capacity to bind to estrogen receptors on hepatocytes and thus regulate expression and activity of some of the many enzymes involved in BAs synthesis [31]. An inhibitory effect of estrogens on BAs transport within the liver has also been described, thus ultimately impairing the conjugation pathway [32].

Another important aspect that emerged from our study is that glycine-conjugated BAs levels, especially GCA, were significantly lower in pubertal compared to pre-pubertal phase. Total BAs concentration also tended to be lower in pubertal subgroup compared to the pre-pubertal cohort, though this difference did not reach statistical significance in the present study. Jahnel et al. [33] observed that total BA levels continuously decrease in older children and adolescents, and that the composition of BAs also depends on the age during the first decade, whilst the BAs profile becomes similar to the adulthood in children aged 11 years or older [33]. This seems reasonable considering that the conjugating activity decreases in parallel with the age, and the ratio between conjugated BAs and total BAs is >90% in infants under 1 year of age [34].

Differences in microbiota between pre-pubertal and pubertal children [35] also have a considerable impact on BA composition [36], since of intestinal microbiota deconjugation increases with age [37]. In addition to the effect on deconjugation, the gut microbiota plays a central role in the metabolism of BAs by also regulating dehydroxylation, dehydrogenation, and epimerization to convert primary BAs into unconjugated secondary and free BAs [37]. Moreover, since microbiota affects energy metabolism and host lipid metabolism, leading to decreased triglyceride levels in serum but increased levels in adipose and liver tissue [38], microbiota influences the synthesis of BAs from cholesterol in the liver.

No differences could be found in the concentration of BAs between hypertensive and normotensive obese children and adolescents in our study. Although sporadic evidence in animal models suggested a possible antihypertensive role for BAs administration [39,40], no study demonstrated the existence of a significant relationship between BAs and BP in humans to the best of our knowledge.

The main finding of this study is that the total concentration of BAs, primary CA and primary glycine- (i.e., GCA and GCDCA) and taurine-conjugated BAs (i.e., TCA, taurocholic acid), seem to be significantly higher in children and adolescents with steatosis compared to those without. In particular, primary glycine conjugated BAs and primary taurine-conjugated cholic acid were found to be higher in young subjects with steatosis than in those without. Moreover, glycine-conjugated BAs were also found to be significantly correlated with AST and ALT, which are well-known markers of liver damage.

Similar changes in circulating BAs have been earlier described by Puri and colleagues [41] in 86 adults (24 controls, 25 NAFL, and 37 NASH; mean BMI, 31.9 kg/m^2^). Conjugated primary BAs (GCA and GCDCA) were found to be significantly higher in NASH and NAFLD patients than in healthy controls, especially in NASH.

The increase of circulating levels of primary BAs and in particular conjugated primary BAs in children with steatosis may entail three different mechanisms, i.e., increased synthesis of primary BAs, decreased conversion to secondary BAs in the intestine or, finally, decreased bile excretion.

Interestingly, Puri et al. analyzed the expression of cytochrome 7α1 hydroxylase (Cyp7A) [41], the enzyme involved in initiation of classic BAs biosynthetic pathway [42], and found elevated Cyp7A expression level in both NAFLD and NASH patients, thus suggesting that BAs synthesis may be conserved in these patients.

In favour of the third mechanism (i.e., decrease in bile excretion), comes the observation that increased glycine conjugated BAs concentrations (especially primary glycine-conjugated BAs), has been previously described in obstructive cholangiopathy and biliary atresia [43,44]. In these conditions, the excretion of BAs into the intestine is reduced, thus leading to lower generation of deconjugated and secondary BAs [45].

Another important aspect to consider is that, compared with CDCA, CA and its conjugates exhibit an overall lower potency as farnesoid-x-receptor (FXR) agonist [46], which plays a key regulatory role in glucose homeostasis [47]. Accordingly, FXR-null mice develop severe fatty liver with elevated serum glucose and impaired glucose and insulin tolerance [47].

The increases concentration of CA and its conjugates in children with steatosis may hence ultimately foster insulin resistance, thus supporting the hypothesis that abnormal BAs profile may have a role in the pathogenesis and severity of liver disease starting from the childhood.

Unexpectedly, we failed to observe a significant association between severity of obesity (assessed as BMI and waist/height ratio) and total BAs values or their profile. However, our results are in keeping with those reported by Higgins et al., in 30 obese and 15 normal weight children [48]. In this previous study, postprandial, but not fasting, total BAs values were found to be significantly lower in obese compared to normal weight adolescents, whilst postprandial BAs profiles were also differently distributed. Interestingly, post-prandial circulating BAs have been reported as biomarkers of appetite [49], as their concentration correlate with that of key appetite-related gut hormones [50,51].

We have finally explored the effect of BAs on vascular elasticity and morphology, showing that total glycine-conjugate acids were positively correlated with cDC (10^−3^-Kpa and percentile), a correlation that remained significant even after adjusting for sex, age, pubertal stage, BMI, and AST. Among the single BAs, GUDCA was positive correlated with cDC; GDCA and TDCA were also found to be positively correlated with cIMT, but these associations were no longer significant after adjusting for sex, age, pubertal stage, BMI, and AST.

A decrease of common carotid artery dispensability (i.e., increased local arterial stiffness) and enhanced cIMT are considered suitable indicators of early subclinical atherosclerosis and future CVD [52,53,54]. Some longitudinal studies showed that children with obesity and increased cIMT are at higher risk of CVD in adulthood [55,56].

BAs, after intestinal metabolism by the microbiota, partly enter systemic circulation and reach a series of target organs other than the liver, where they act as natural ligands for several receptors. The two most important of such receptors are the G protein-coupled bile acid receptor 1 (GPBAR1) and the previously mentioned FXR, which are largely expressed in different cells (nonparenchymal liver cells, enterocytes, endocrine intestinal cells and neurons). GPBAR1 is also expressed in vascular endothelial and smooth muscle cells, and this would suggest a role for this receptor in regulating systemic circulation [57]. BAs also interact with large-conductance Ca^2+^-activated K+ (BKCa) channels that regulate arterial tone and induces vessel relaxation [58]. In the study by Pak et al. [59], the authors showed that the vasodilatory effect of BAs in mice is mediated by their Ca^2+^-antagonistic property. In another study, Khurana sand colleagues reported that deoxycholylglycine (DCG), a conjugated secondary bile acid, displayed vasodilatory effect in mice by impairing Ca^2+^ sensitivity of small resistance arteries and reducing Rho kinase activator (ROCK)-induced vasoconstriction, finally inhibiting ROCK activation as a consequence of attenuated RhoA membrane translocation [60]. Although BAs can act as vasoactive ligands regulating vascular tone in physiological conditions [61], in some diseases such as cirrhosis BAs largely shift from the enterohepatic to the systemic circulation, then promoting exaggerated vasodilation, reducing systemic vascular resistance and finally leading to a hyperdynamic circulation [62].

FXR ligands also play a role in inhibiting vascular smooth muscle cell inflammation and migration, thus reducing the atherosclerotic process [63]. Although this beneficial effect has been described as especially dependent from taurine-conjugated BAs [64,65], other BAs may also play a role.

The observation that BAs act as hormones by activating different receptors but also by activating receptor-independent cellular signalling cascades and regulating epigenetic machinery led to consider them as novel promising therapeutic agents in different diseases [66]. Even if bile acid therapy based on the use of bile acid agonists or bile acid antagonists has been mainly applied in adults, it could have potential clinical implications even in children.

The present study has several limitations. First, only fasting BAs concentrations were measured in our study, and some components of BAs were under the LOQ. Second, due to the large number of investigations performed and to the difficulty of obtaining parental consent to carry out additional tests in children, the sample size was quite limited. Third, we did not have a population of healthy controls matched by sex and age and could not conduct a case-control study. Finally, the cross-sectional design of this study would not allow to infer any causal relationship between the measured variables.

In conclusion, we observed significant differences in the circulating BAs profile in a group of obese children and adolescents, depending on sex, pubertal stage and presence of steatosis. An association between some BAs and vascular damage indices was also noted, thus paving the way to future studies aimed at reproducing and validating our preliminary findings.

## Figures and Tables

**Table 1:** General characteristics of the 69 obese children and adolescents divided according to sex and pubertal stage.

	Total Population *n* = 69	Male *n* = 39	Female *n* = 30		Pre-Pubertal Stage *n* = 37	Post-Pubertal Stage *n* = 32	
	Median (IQR)	Median (IQR)	Median (IQR)	*p*-Value *	Median (IQR)	Median (IQR)	*p*-Value *
Age, ys	11 (10–13)	11.0 (10.0–14.0)	11.0 (9.0–13.0)	n.s.	10 (9.0–11.0)	13.0 (12.0–15.0)	<0.0001
BMI, kg/m^2^ BMI, percentile	28.9 (26–31.6) 98.4 (97.5–99.2)	28.4 (25.5–31.0 98.3 (97.2–99.1	29.3 (26.0–32.8) 98.7 (97.7–99.3)	n.s.	28.2 (2532–30.5) 98.9 (97.8–99.3)	29.9 (26.3–33.5) 98.0 (96.6–98.9)	n.s. 0.036
Office SBP, mmHg Office SBP, percentile	112 (104.5–122) 65.6 (49.6–90.1)	115.0 (110.0–122.0) 70.5 (50.2–90.3)	110.0 (104.0–121.0) 63.3 (48.2–91.0)	n.s. n.s.	110.0 (104.0–120.0) 65.6 (48.8–89.4)	116.0 (110.0–130.0) 67.5 (50.2–67.5)	0.024 n.s.
Office DBP, mmHg Office DBP, percentile	70.0 (68,0–77,3) 73.5 (57.2–87.1)	73.0 (68.0–80.0) 74.1 (55.2–90.6)	70.0 (64.8–72.8) 72.9 (59.5–82.2)	n.s. n.s.	70.0 (66.5–74.0) 76.1 (68.5–87.1)	70.0 (68.0–80.0) 67.1 (49.5–91.2)	n.s. n.s.
24-h SBP, mmHg 24-h SBP, percentile	116.0 (110.0–120.0) 62.8 (39.9–81.4)	118.0 (114.0–121.0) 68.2 (53.1–81.2)	112.0 (106.5–116.0) 51.8 (28.9–82.4)	0.001 n.s.	115.0 (109.3–121.0) 68.2 (50.1–82.1)	117.0 (110.0–120.0) 56.7 (33.8–77.0)	n.s. 0.010
Daytime SBP, mmHg Daytime SBP, percentile	119.0 (113.0–125.0) 61.8 (34.4–77.4)	123.5 (116.0–128.0) 71.6 (44.2–82.9)	115.0 (110.0–120.0) 53.9 (20.0–70.9)	0.001 0.014	117.5 (113.5–124.0) 66.4 (37.5–81.2)	120.0 (113.0–125.0) 59.5 (28.8–74.6)	n.s. n.s.
Nighttime SBP, mmHg Nighttime SBP, percentile	107.0 (104.0–113.0) 75.5 (61.8–93.8)	108.0 (106.0–113.0) 77.0 (63.6–90.3)	104.5 (100.0–113.5) 75.2 (56.4–95.1)	0.001 0.004	107.5 (104.0–113.0) 86.2 (66.5–95.4)	107.0 (103.0–113.0) 70.9 (44.2–87.5)	n.s. n.s.
24-h DBP, mmHg 24-h DBP, percentile	66.0 (62.0–69.0) 41.2 (23.5–63.0)	68.0 (64.0–70.3) 55.0 (26.8–71.3)	64.0 (61.0–67.0) 33.9 (15.8–47.0)	0.001 0.004	67.0 (63.3–70.0) 48.9 (26.1–70.0)	65.0 (62.0–69.0) 36.0 (14.7–60.5)	n.s. n.s.
Daytime DBP, mmHg Daytime DBP, percentile	70.0 (65.0–74.0) 35.5 (15.8–62.0)	71.0 (67.8–76.0) 41.9 (22.7–74.1)	67.0 (64.0–71.0) 22.7 (12.0–40.4)	0.030 0.003	70.0 (67.0–73.8) 36.3 (21.3–60.8)	68.0 (65.0–74.0) 22.7 (11.2–62.2)	n.s. n.s.
Nighttime DBP, mmHg Nighttime DBP, percentile	58.0 (56.0–62.0) 68.7 (53.6–86.4)	61.0 (57.0–64.0) 79.3 (57.0,90.7)	57.0 (54.3–59.0) 64.6 (48.8–74.4)	0.005 n.s.	59.0 (56.3–64.0) 71.5 (57.7–90.6)	58.0 (56.0–61.0) 66.1 (50.7–79.3)	n.s. n.s.
cIMT, mm cIMT, percentile	0.46 (0.40,0.50) 94.4 (68.2–99.3)	0.47 (0.42–0.51 98.8 (80.3–99.7)	0.42 (.40-.47) 84.1 (62.0–97.0	0.04 0.02	0.46 (0.41–0.51) 98.8 (80.4–99.7)	0.44 (0.40–0.48) 88.2 (61.5–97.7)	n.s. 0.026
cDC, 10^−3^-Kpa cDC, percentile	40.7 (34.7–47.0) 11.7 (2.6–25.5)	39.7 (33.5–46.4) 6.4 (2.4–23.2)	43.8 (35.8–48.5) 13.2 (3.1–27.0)	n.s. n.s.	43.3 (35.9–49.7) 11.9 (4.0–27.9)	39.7 (32.7–44.2) 1.09 (2.1–20.9)	n.s. n.s.
Total cholesterol, mg/dL	160.5 (134.8–188.8)	160.0 (140.0–185.5)	162.0 (134.0–191.0)	n.s.	165.0 (139.0–183.0)	153.0 (134.0–191.0)	n.s.
HDL, mg/dL	49.5 (42.5–56.0)	49.0 (39.0–52.0)	52 (45.0–59.0)	0.044	50.0 (41.0–55.0)	49.0 (43.0–59.0)	n.s.
Triglycerides, mg/dL	79.0 (54.0–100.0)	80.5 (55.5–112.0)	73.0 (52.0–100.0)	n.s.	85.5 (66.8–110.5)	69.0 (49.0–98.0)	n.s.
FPG, mg/dL	86.0 (82.8–91.0)	88.0 (84.3–93.0)	85.0 (78.5–88.0)	0.013	88.0 (83.8–90.3)	86.0 (82.0–93.3)	n.s.
Fasting insulin, μU/mL	18.2 (12.5–28.0)	18.9 (12.4–27.8)	17.8 (13.5–28.2)	n.s.	17.7 (11.9–24.7)	20.7 (14.3–28.7)	n.s.
ALT, U/L	24.0 (17.3–40.0)	29.0 (20.5–45.5)	19.0 (15.0–25.0)	0.007	23.5 (18.3–44.3)	24.5 (16.3–35.8)	n.s.
AST, U/L	26.0 (26.0–33.0)	27.5 (23.8–35.5)	21.5 (18.3–28.0)	0.003	27.0 (22.0–34.0)	24.0 (19.5–30.0)	n.s.
GGT, U/L	14.0 (12.0–20.6)	16.0 (13.0–22.0)	13.0 (10.0,18.0)	0.022	14.0 (12.0–18.0)	14.0 (11.0–22.5)	n.s.

* Wilcoxon–Mann Whitney U test; ALT, alanine aminotransferase; AST, aspartate aminotransferase; BMI, body mass index, cIMT, carotid intima-media thickness; cDC, carotid distensibility coefficient; FPG, fasting plasma glucose; GGT, gamma-glutamyltransferase; HDL, HDL cholesterol; SBP, systolic blood pressure; DBP, diastolic blood pressure.

**Table 2:** Bile acids concentrations in the 69 obese children and adolescents divided according to sex and pubertal stage.

	Total Population *n* = 69	Male *n* = 39	Female *n* = 30		Pre-Pubertal Stage *n* = 37	Pubertal Stage *n* = 32	
	Median (IQR)	Median (IQR)	Median (IQR)	*p*-Value *	Median (IQR)	Median (IQR)	*p*-Value *
Single BAs							
CA, ng/mL	27.3 (11.5–76.5)	29.3 (11.7–66.7)	26.7 (10.9–90.8)	n.s.	26.0 (17.7–77.7)	31.9 (8.5–66.7)	n.s.
CDCA, ng/mL	82.5 (34.3–182.5)	82.5 (34.6–163.9)	78.7 (27.8–202.4)	n.s.	88.7 (37.1–213.1)	73.6 (20.2–129.6)	n.s.
DCA, ng/mL	78.7 (36.8–78.7)	78.6 (47.6–139.9)	75.7 (17.3–162.6)	n.s.	77.3 (39.5–173.5)	80.3 (22.5–139.8)	n.s.
UDCA, ng/mL	19.6 (4.5–37.9)	19.6 (5.4–50.6)	20.8 (3.5–37.0)	n.s.	19.6 (4.5–46.3)	19.0 (4.1–33.6)	n.s.
GCDCA, ng/mL	139.4 (61.4–221.2)	183.5 (91.5–306.7)	107.3 (38.3–166.1)	**0.006**	165.2 (87.8–275.2)	115.4 (50.3–185.8)	n.s.
GDCA, ng/mL	27.9 (11.4–55.6)	39.3 (18.9–87.9)	16.6 (10.8–47.0)	**0.010**	39.3 (11.1–81.9)	23.7 (12.0–52.8)	n.s.
GUDCA, ng/mL	35.0 (16.4–49.8)	41.4 (27.0–70.1)	19.5 (11.8–40.3)	**0.007**	39.7 (21.5–76.0)	30.0 (15.4–42.2)	n.s.
GCA, ng/mL	39.6 (23.7–71.6)	47.0 (28.3–73.1)	29.9 (13.2–68.4)	**0.03**	48.1 (27.8–80.9)	32.2 (14.5–51.2)	**0.03**
TCA, ng/mL	3.5 (3.5–7.7)	5.6 (3.5–9.8)	3.5 (3.5–7.3)	n.s.	5.5 (3.5–9.9)	3.5 (3.5–7.0)	n.s.
TCDCA, ng/mL	17.1 (11.6–29.7)	20.1 (12.5–32.8)	16.0 (5.8–24.9)	n.s.	20.1 (11.8–33.7)	16.5 (7.9–23.5)	n.s.
TDCA, ng/mL	3.5 (3.5–8.5)	3.5 (3.5–14.0)	3.5 (3.5–6.6)	n.s.	3.5 (3.5–14.0)	3.5 (3.5–6.9)	n.s.
HDCA, ng/mL	3.5 (3.5–7.5)	3.5 (3.5–8.1)	3.5 (3.5–7.4)	n.s.	3.5 (3.5–8.9)	3.5 (3.5–5.8)	n.s.
Classes of BAs							
Total BAs, ng/mL	600.3 (332.2–954.5)	670.1 (413.8–1047.6)	510.8 (245.4–847.1)	n.s.	676.7 (365.9–1073.5)	504.3 (262.8–795.2)	n.s.
Primary BAs, ng/mL	112.0 (50.8–265.4)	110.9 (54.9–245.2)	113.4 (44.4–295.5)	n.s.	114.7 (57.1–290.7)	102.4 (40.7–204.3)	n.s.
Secondary BAs, ng/mL	97.3 (59.0–214.7)	100.6 (66.7–233.2)	90.4 (46.8–212.2)	n.s.	97.3 (66.6–226.5)	99.4 (49.1–183.3)	n.s.
Glycine-conjugated BAs, ng/mL	242.1 (109.8–330.6)	271.5 (164.7–406.8)	161.9 (79.5–275.3)	**0.005**	276.2 (151.1–403.7)	190.4 (87.4–269.4)	**0.02**
Taurine-conjugated BAs, ng/mL	9.1 (7.1–16.6)	10.9 (7.1–23.7)	7.9 (7.1–12.2)	n.s.	10.9 (7.1–24.0)	7.8 (7.1–13.9)	n.s.

* Wilcoxon—Mann Whitney U test. Significant values are in bold (*p* < 0.05).

**Table 3:** Bile acids concentrations in participants with and w/o steatosis.

	Non-Steatosis *n* = 29	Steatosis *n* = 33	
	Median (IQR)	Median (IQR)	*p*-Value *
Single BAs			
CA, ng/mL	17.3 (8.3–51.8)	40.7 (19.4–106.6)	**0.019**
CDCA, ng/mL	79.0 (15.5–116.6)	92.2 (35.8–313.7)	n.s.
DCA, ng/mL	80.6 (40.4–135.7)	75.3 (30.9–145.7)	n.s.
UDCA, ng/mL	16.9 (3.5–28.2)	27.7 (5.6–55.8)	n.s.
GCDCA, ng/mL	104.7 (50.1–170.0)	187.0 (93.9–325.7)	**0.009**
GDCA, ng/mL	19.9 (11.4–48.5)	36.6 (10.9–87.1)	n.s.
GUDCA, ng/mL	20.7 (11.7–39.8)	42.5 (28.0–87.4)	n.s.
GCA, ng/mL	30.5 (13.9–42.5)	59.9 (27.8–90.1)	**0.01**
TCA, ng/mL	3.5 (3.5–6.1)	6.0 (3.5–11.0)	**0.04**
TCDCA, ng/mL	14.6 (6.7–22.8)	23.2 (12.2–37.8)	n.s.
TDCA, ng/mL	3.5 (3.5–5.1)	3.5 (3.5–15.2)	n.s.
HDCA, ng/mL	3.5 (3.5–7.5)	3.5 (3.5–6.4)	n.s.
Classes of BAs			
Total BAs, ng/mL	454.6 (254.9–664.2)	796.6 (472.0–1278.0)	**0.004**
Primary BAs, ng/mL	96.3 (31.3–155.3)	112.9 (52.9–404.0)	n.s.
Secondary BAs, ng/mL	91.3 (51.1–163.9)	100.6 (59.0–219.6)	n.s.
Glycine-conjugated BAs, ng/mL	177.6 (82.2–275.6)	288.3 (136.7–485.5)	**0.004**
Taurine-conjugated BAs, ng/mL	7.1 (7.1–11.8)	14.3 (7.1–27.2)	n.s.

* Wilcoxon–Mann Whitney U test. Significant values are in bold (*p* < 0.05).

**Table 4:** Correlations between bile acids (Bas) and age, body mass index (BMI), waist/height ratio, and liver enzymes.

	Age	BMI	Waist/Height Ratio	ALT	AST	GGT
CA	0.023	0.037	0.063	0.170	0.099	0.199
CDCA	−0.006	0.006	0.135	0.159	0.018	0.075
DCA	0.086	−0.006	−0.022	0.147	0.070	0.143
UDCA	−0.060	−0.087	0.118	0.186	0.039	0.214
GCDCA	−0.061	−0.022	0.098	0.193	0.322 *	0.098
GDCA	0.118	0.020	0.051	0.255 *	0.276 *	0.208
GUDCA	−0.143	−0.126	0.095	0.282 *	0.320 *	0.227
GCA	−0.097	0.078	0.165	0.285 *	0.349 **	0.154
TCA	−0.067	−0.010	0.046	0.100	0.134	0.113
TCDCA	−0.077	−0.060	0.084	0.092	0.183	−0.007
TDCA	−0.034	0.035	0.081	0.208	0.212	0.162
HDCA	−0.114	−0.036	−0.068	−0.064	0.061	−0.107
Total BAs	−0.090	−0.059	0.156	0.238	0.233	0.154
Primary BAs	−0.010	0.005	0.114	0.160	0.058	0.107
Secondary BAs	0.004	−0.074	0.021	0.190	0.091	0.159
Glycine-conjugated BAs	−0.141	−0.068	0.137	0.249 *	0.352 **	0.126
Taurine-conjugated BAs	−0.054	0.014	0.103	0.167	0.192	0.174

* *p* < 0.05; ** *p* < 0.001. Significant values are in bold.

**Table 5:** Correlations between BAs and markers of subclinical vascular damage.

	cIMT, mm	cIMT, Percentile	cDC, 10^−3^-Kpa	cDC, Percentile
CA	0.219	0.217	0.167	0.192
CDCA	0.108	0.135	0.184	0.197
DCA	0.208	0.163	0.046	0.018
UDCA	0.005	0.041	0.242	0.243
GCDCA	0.055	0.090	0.244	0.251 *
GDCA	0.310 **	0.270 *	0.073	0.105
GUDCA	0.007	0.072	0.323 *	0.309 *
GCA	0.171	0.201	0.143	0.140
TCA	0.085	0.089	0.038	0.035
TCDCA	0.017	0.004	0.266 *	0.267 *
TDCA	0.272 *	0.254 *	0.031	0.046
HDCA	0.162	0.164	0.097	0.106
Total BAs	0.159	0.191	0.241	0.249
Primary BAs	0.130	0.154	0.207	0.220
Secondary BAs	0.182	0.169	0.035	0.065
Glycine-conjugated BAs	0.071	0.129	0.268 *^,#^	0.264 *^,#^
Taurine- conjugated BAs	0.174	0.160	0.081	0.089

* *p* < 0.05; ^#^ In linear regression, this association remained significant after adjustment for sex, age, pubertal stage, BMI, and aspartate aminotransferase (AST). Significant values are in bold.

## Abbreviations

ALT	alanine aminotransferase
AST	aspartate aminotransferase
BAs	bile acids
BARs	bile acid-activated receptors
BMI	body mass index
BP	blood pressure
CA	cholic acid
cDC	common carotid artery distensibility
cIMT	carotid intima media thickness
CDCA	chenodeoxycholic acid
DCA	deoxycholic acid
DCG	deoxycholylglycine
FPG	fasting plasma glucose
GCA	glycocholic acid
GCDCA	glycochenodeoxycholic acid
GDCA	glycodeoxycholic acid
GGT	gamma-glutamyl transferase
GLCA	glycolithocholic acid
GUDCA	glycoursodeoxycholic acid
HDL	high density lipoprotein
HPLC	high performance liquid chromatography
IQR	interquartile range
LCA	lithocholic acid
LOQ	limit of quantitation
NAFLD	non-alcoholic fatty liver disease
PP	pulse pressure
TCA	taurocholic acid
TCDCA	taurochenodeoxycholic acid
TDCA	taurodeoxycholic acid
TLCA	taurolithocholic acid
TUDCA	tauroursodeoxycholic acid
UDCA	ursodeoxycholic acid
US	ultrasonography

## Appendix A

**Table A1:** General characteristics of children and adolescents with and without liver steatosis.

	W/o Steatosis *n* = 29	With Steatosis *n* = 33	
	Median (IQR)	Median (IQR)	*p*-Value *
Age, y	11.0 (9.5–13.0)	11.0 (10.0–13.5)	n.s.
BMI, kg-m^2^ BMI, percentile	28.2 (25.2–31.8) 98.2 (97.3–99.2)	29.5 (26.2–31.6) 98.8 (97.6–99.3)	n.s.
Office SBP, mmHg Office SBP, percentile	112.0 (104.0–129.0) 70.5 (49.4–96.5)	114.0 (104.0–122.0) 63.0 (46.4–89.1)	n.s.
Office DBP, mmHg Office DBP, percentile	70.0 (68.0–79.0) 76.3 (61.4–91.3)	70.0 (66.0–79.0) 70.3 (52.1–86.1)	n.s.
24-h SBP, mmHg 24-h SBP, percentile	116.0 (108.0–121.0) 59.7 (38.2–87.3)	116.0 (110.0–120.0) 46.4 (62.8–77.0)	n.s.
Daytime SBP, mmHg Daytime SBP, percentile	120.0 (113.5–126.0) 61.4 (38.7–88.3)	120.0 (113.0–125.0) 70.0 (67.0–74.0)	n.s.
Nighttime SBP, mmHg Nighttime SBP, percentile	107.0 (102.0–113.0) 75.2 (55.4–75.2)	107.0 (104.8–113.3) 78.8 (65.5–87.6)	n.s.
24-h DBP, mmHg 24-h DBP, percentile	64.0 (62.0–70.0) 34.9 (14.8–68.5)	67.0 (64.0–69.0) 47.0 (25.6–61.8)	n.s.
Daytime DBP, mmHg Daytime DBP, percentile	69.0 (64.0–74.5) 29.5 (12.2–59.9)	70.0 (67.0–74.0) 35.5 (18.7–62.6)	n.s.
Nighttime DBP, mmHg Nighttime DBP, percentile	57.0 (54.0–64.0) 63.8 (47.6–90.6)	59.0 (56.8–62.5) 72.5 (57.4–86.8)	n.s.
cIMT, mm cIMT, percentile	0.43 (0.40–0.50) 88.2 (61.2–99.1)	0.46 (0.40–0.48) 94.3 (74.6–99.1)	n.s.
cDC, 10^−3^-Kpa cDC, percentile	41.2 (33.6–49.5) 11.9 (2.2–26.3)	39.9 (35.1–46.9) 10.9 (2.7–20.9)	n.s.
Total cholesterol, mg/dL	169.5 (150.5–191.0)	153.0 (133.3–173.0)	n.s.
HDL, mg/dL	53.0 (43.0–59.0)	49.0 (39.0–52.0)	n.s.
Triglycerides, mg/dL	81.5 (55.5–120.0)	78.5 (55.8–106.5)	n.s.
Fasting plasma glucose, mg/dL	88.0 (82.0–93.0)	86.0 (83.3–90.8)	n.s.
Fasting Insulin, μU/mL	18.5 (11.1–27.3)	18.5 (14.0–28.1)	n.s.
ALT, U/L	20.0 (16.8–25.0)	30.0 (19.5–48.0)	**0.039**
AST, U/L	23.0 (20.0–27.0)	28.0 (22.5–36.5)	n.s.
GGT, U/L	13.0 (10.8–14.5)	16.5 (13.0–26.5)	**0.005**

Abdomen ultrasonography of 7 children was not available. * Wilcoxon-Mann Whitney U test. Significant values are in bold (*p* < 0.05). ALT, alanine aminotransferase; AST, aspartate aminotransferase; BMI, body mass index, cIMT, carotid intima-media thickness; cDC, carotid distensibility coefficient; GGT, gamma-glutamyltransferase; HDL, HDL cholesterol; SBP, systolic blood pressure; DBP, diastolic blood pressure.

**Table A2:** Bile acids concentrations in normotensive and hypertensive obese children and adolescents.

	Normotensive *n* = 50	Hypertensive *n* = 17	
	Median (IQR)	Median (IQR)	*p*-Value *
Single BAs			
CA, ng/mL	25.3 (10.8–69.3)	27.3 (11.6–76.6)	n.s.
CDCA, ng/mL	89.2 (36.4–203.2)	55.4 (22.0–117.0)	n.s.
DCA, ng/mL	72.6 (23.4–152.1)	87.8 (53.0–188.0)	n.s.
UDCA, ng/mL	21.8 (6.3–44.2)	8.6 (3.5–29.0)	n.s.
GCDCA, ng/mL	139.6 (70.8–199.0)	104.7 (45.2–249.3)	n.s.
GDCA, ng/mL	27.8 (11.3–55.0)	35.1 (12.5–76.1)	n.s.
GUDCA, ng/mL	39.1 (18.3–54.3)	29.3 (11.3–47.5)	n.s.
GCA, ng/mL	40.8 (21.6–70.8)	36.2 (19.8–68.5)	n.s.
TCA, ng/mL	5.3 (3.5–7.6)	3.5 (3.5–8.6)	n.s.
TCDCA, ng/mL	17.8 (11.7–29.8)	15.7 (10.1–32.3)	n.s.
TDCA, ng/mL	3.5 (3.5–7.5)	5.0 (3.5–12.0)	n.s.
HDCA, ng/mL	3.5 (3.5–6.2)	3.5 (3.5–10.3)	n.s.
Classes of BAs			
Total BAs, ng/mL	614.3 (386.0–899.7)	557.2 (236.7–954.5)	n.s.
Primary BAs, ng/mL	113.4 (55.3–222.8)	96.3 (43.3–190.6)	n.s.
Secondary BAs, ng/mL	91.2 (55.2–212.4)	97.3 (59.3–212.6)	n.s.
Glycine-conjugated BAs, ng/mL	243.3 (129.0–323.8)	177.6 (93.1–330.6)	n.s.
Taurine-conjugated BAs, ng/mL	9.0 (7.1–15.6)	10.8 (7.1–22.4)	n.s.

Blood pressure measures of 2 children were not available. ***** Wilcoxon-Mann Whitney U test. BAs, bile acids; CA, cholic acid; CDCA, chenodeoxycholic acid; DCA, deoxycholic acid; DCG, deoxycholylglycine; GCA, glycocholic acid; GCDCA, glycochenodeoxycholic acid; GDCA, glycodeoxycholic acid; GLCA, glycolithocholic acid; GUDCA, glycoursodeoxycholic acid; IQR, interquartile range; LCA, lithocholic acid; TCA, taurocholic acid; TCDCA, taurochenodeoxycholic acid; TDCA, taurodeoxycholic acid; TLCA, taurolithocholic acid; TUDCA, tauroursodeoxycholic acid; UDCA, ursodeoxycholic acid.
